# Supplementary material for: Role and mechanism of NCAPD3 in promoting malignant behaviors in gastric cancer
Source: Front Pharmacol. 2024 Apr 22;15:1341039. doi: 10.3389/fphar.2024.1341039 (PMC11070777; doi:10.3389/fphar.2024.1341039)
Supplement: Supplementary file 11 [file DataSheet2.ZIP › GSEA/Oncogenic signature/my_analysis.Gsea.1599461962066/ESC_J1_UP_LATE.V1_DN.html]

Details for gene set ESC\_J1\_UP\_LATE.V1\_DN[GSEA]

|  || Dataset | filtered\_dataset.sample\_info.cls#WT\_versus\_NCAPD3\_MUT |
| Phenotype | sample\_info.cls#WT\_versus\_NCAPD3\_MUT |
| Upregulated in class | WT |
| GeneSet | ESC\_J1\_UP\_LATE.V1\_DN |
| Enrichment Score (ES) | 0.37138733 |
| Normalized Enrichment Score (NES) | 1.7515038 |
| Nominal p-value | 0.02124183 |
| FDR q-value | 0.22261527 |
| FWER p-Value | 0.58 |
Table: GSEA Results Summary

  

Fig 1: Enrichment plot: ESC\_J1\_UP\_LATE.V1\_DN      
 Profile of the Running ES Score & Positions of GeneSet Members on the Rank Ordered List

  

| SYMBOL | TITLE | RANK IN GENE LIST | RANK METRIC SCORE | RUNNING ES | CORE ENRICHMENT || 1 | 55170 | PRMT6 | 32 | 0.981 | 0.0534 | Yes |
| 2 | 55781 | RIOK2 | 135 | 0.763 | 0.0392 | Yes |
| 3 | 80312 | TET1 | 139 | 0.757 | 0.0960 | Yes |
| 4 | 23590 | PDSS1 | 175 | 0.701 | 0.1253 | Yes |
| 5 | 55599 | RNPC3 | 227 | 0.652 | 0.1393 | Yes |
| 6 | 54865 | GPATCH4 | 261 | 0.622 | 0.1640 | Yes |
| 7 | 91860 | CALML4 | 289 | 0.600 | 0.1912 | Yes |
| 8 | 2997 | GYS1 | 376 | 0.548 | 0.1718 | Yes |
| 9 | 54517 | PUS7 | 388 | 0.544 | 0.2062 | Yes |
| 10 | 5245 | PHB | 481 | 0.486 | 0.1776 | Yes |
| 11 | 11060 | WWP2 | 503 | 0.471 | 0.1992 | Yes |
| 12 | 26049 | FAM169A | 533 | 0.455 | 0.2137 | Yes |
| 13 | 26164 | MTG2 | 585 | 0.433 | 0.2106 | Yes |
| 14 | 23195 | MDN1 | 596 | 0.426 | 0.2366 | Yes |
| 15 | 55622 | TTC27 | 620 | 0.412 | 0.2521 | Yes |
| 16 | 643836 | ZFP62 | 639 | 0.405 | 0.2707 | Yes |
| 17 | 51728 | POLR3K | 646 | 0.401 | 0.2976 | Yes |
| 18 | 2021 | ENDOG | 648 | 0.400 | 0.3280 | Yes |
| 19 | 6905 | TBCE | 652 | 0.399 | 0.3570 | Yes |
| 20 | 79832 | QSER1 | 675 | 0.389 | 0.3714 | Yes |
| 21 | 81627 | TRMT1L | 745 | 0.353 | 0.3491 | No |
| 22 | 10492 | SYNCRIP | 779 | 0.334 | 0.3513 | No |
| 23 | 84823 | LMNB2 | 833 | 0.293 | 0.3359 | No |
| 24 | 8835 | SOCS2 | 840 | 0.275 | 0.3530 | No |
| 25 | 6789 | STK4 | 915 | -0.345 | 0.3264 | No |
| 26 | 9314 | KLF4 | 972 | -0.389 | 0.3162 | No |
Table: GSEA details [plain text format]

  

Fig 2: ESC\_J1\_UP\_LATE.V1\_DN      
 Blue-Pink O' Gram in the Space of the Analyzed GeneSet

  

Fig 3: ESC\_J1\_UP\_LATE.V1\_DN: Random ES distribution      
 Gene set null distribution of ES for **ESC\_J1\_UP\_LATE.V1\_DN**
